# Prospective association of the infant gut microbiome with social behaviors in the ECHO consortium

**DOI:** 10.1186/s13229-024-00597-2

**Published:** 2024-05-17

**Authors:** Hannah E. Laue, Kevin S. Bonham, Modupe O. Coker, Yuka Moroishi, Wimal Pathmasiri, Susan McRitchie, Susan Sumner, Anne G. Hoen, Margaret R. Karagas, Vanja Klepac-Ceraj, Juliette C. Madan, program collaborators for Environmental influences on Child Health Outcomes, P. B. Smith, K. L. Newby, L. P. Jacobson, D. J. Catellier, R. Gershon, D. Cella, D. Koinis Mitchell, S. Deoni, V. D’Sa

**Affiliations:** 1 Department of Epidemiology, Geisel School of Medicine, Dartmouth College, Hanover, NH USA; 2 Department of Biological Sciences, Wellesley College, 106 Central Street, Wellesley, MA 02481 USA; 3 School of Dental Medicine, Rutgers University, Newark, NJ USA; 4 Department of Nutrition, Nutrition Research Institute, University of North Carolina at Chapel Hill, Chapel Hill, NC USA; 5 Departments of Pediatrics and Psychiatry, One Medical Center Drive, Dartmouth Hitchcock Medical Center, Lebanon, NH 03756 USA

**Keywords:** Microbiome, Autism, Social behavior, Gut metagenome, Gene set enrichment analysis

## Abstract

**Background:**

Identifying modifiable risk factors of autism spectrum disorders (ASDs) may inform interventions to reduce financial burden. The infant/toddler gut microbiome is one such feature that has been associated with social behaviors, but results vary between cohorts. We aimed to identify consistent overall and sex-specific associations between the early-life gut microbiome and autism-related behaviors.

**Methods:**

Utilizing the Environmental influences on Children Health Outcomes (ECHO) consortium of United States (U.S.) pediatric cohorts, we gathered data on 304 participants with fecal metagenomic sequencing between 6-weeks to 2-years postpartum (481 samples). ASD-related social development was assessed with the Social Responsiveness Scale (SRS-2). Linear regression, PERMANOVA, and Microbiome Multivariable Association with Linear Models (MaAsLin2) were adjusted for sociodemographic factors. Stratified models estimated sex-specific effects.

**Results:**

Genes encoding pathways for synthesis of short-chain fatty acids were associated with higher SRS-2 scores, indicative of ASDs. Fecal concentrations of butyrate were also positively associated with ASD-related SRS-2 scores, some of which may be explained by formula use.

**Limitations:**

The distribution of age at outcome assessment differed in the cohorts included, potentially limiting comparability between cohorts. Stool sample collection methods also differed between cohorts. Our study population reflects the general U.S. population, and thus includes few participants who met the criteria for being at high risk of developing ASD.

**Conclusions:**

Our study is among the first multicenter studies in the U.S. to describe prospective microbiome development from infancy in relation to neurodevelopment associated with ASDs. Our work contributes to clarifying which microbial features associate with subsequent diagnosis of neuropsychiatric outcomes. This will allow for future interventional research targeting the microbiome to change neurodevelopmental trajectories.

**Supplementary Information:**

The online version contains supplementary material available at 10.1186/s13229-024-00597-2.

## Background

The connection between the gut microbiome and the brain has important foundational implications in the critical window following birth until early childhood [1, 2]. During the neonatal period through 3 years of age, microbe-microbe and microbe-human interactions are required for the developing innate and adaptive immune systems to achieve competency [3], and the intestinal microbiome plays a critical role in neurodevelopment during this important window [4–6]. For example, brain volume differences between specific-pathogen-free mice and germ-free mice, even when the microbiomes of the germ-free mice are conventionalized after weaning [7]. Signals from the gut microbiome influence neurophysiology and neurodevelopment via vagus nerve activation, endocrine signaling, and impacts on systemic inflammation and neuroimmune cells [8]. Cross-talk between the gut and developing brain is likely bidirectional and primarily driven by microbially-derived metabolites such as short-chain fatty acids (SCFAs) produced by bacteria as byproducts of fermentation of indigestible polysaccharides, which rapidly change during this critical period of microbiome acquisition and neurodevelopment [9, 10]. Microbial metabolites, including SCFAs, are required for proper functioning and maturation of neuroimmune cells, such as microglia, the innate immune cells critical for neurodevelopment and modulation of neuroinflammation [11].

We recently described associations of early-life gut bacteria and their functions with social behavior at preschool age in a single large United States (U.S.) birth cohort study, complementing research from other groups [12–15]. The gut microbiome is a promising modifiable target for interventions aiming to reduce adverse autism spectrum disorder (ASD) behaviors, which impose a significant financial burden due to increased healthcare utilization, special education costs, and loss of income [16–20]. Modification of the intestinal microbiome results in shifts in key metabolites implicated in social behaviors [13] and alteration of the microbiome can result in reduction of ASD-associated behaviors in both animal and human studies of antibiotics and fecal transplants [21–25]. These and other studies have identified gut microbial differences after ASD diagnosis, when amending the microbiome may be less impactful. Furthermore, a recent case–control study suggested that the mechanism of microbiome-ASD associations is via early dietary preferences in children with ASD [26], highlighting the need for research with microbiome characterization prior to the manifestation of dietary preferences. Such prospective studies beginning in the first weeks of life can clarify opportunities for altering the microbiome to optimize neurodevelopmental outcomes.

We aimed to prospectively characterize the association between the microbiome beginning at birth and ASD-related social behaviors during childhood when rapid neurodevelopment lays the foundation for lifelong optimal health outcomes. Leveraging the resources of the Environmental influences on Child Health Outcomes (ECHO) consortium funded by the National Institutes of Health, we performed a multi-center investigation to identify prospective associations between the gut microbiome and autism-related social behaviors and to determine whether these associations were consistent across cohorts and ages.

## Methods

### Cohort descriptions

**Fig. 1:**
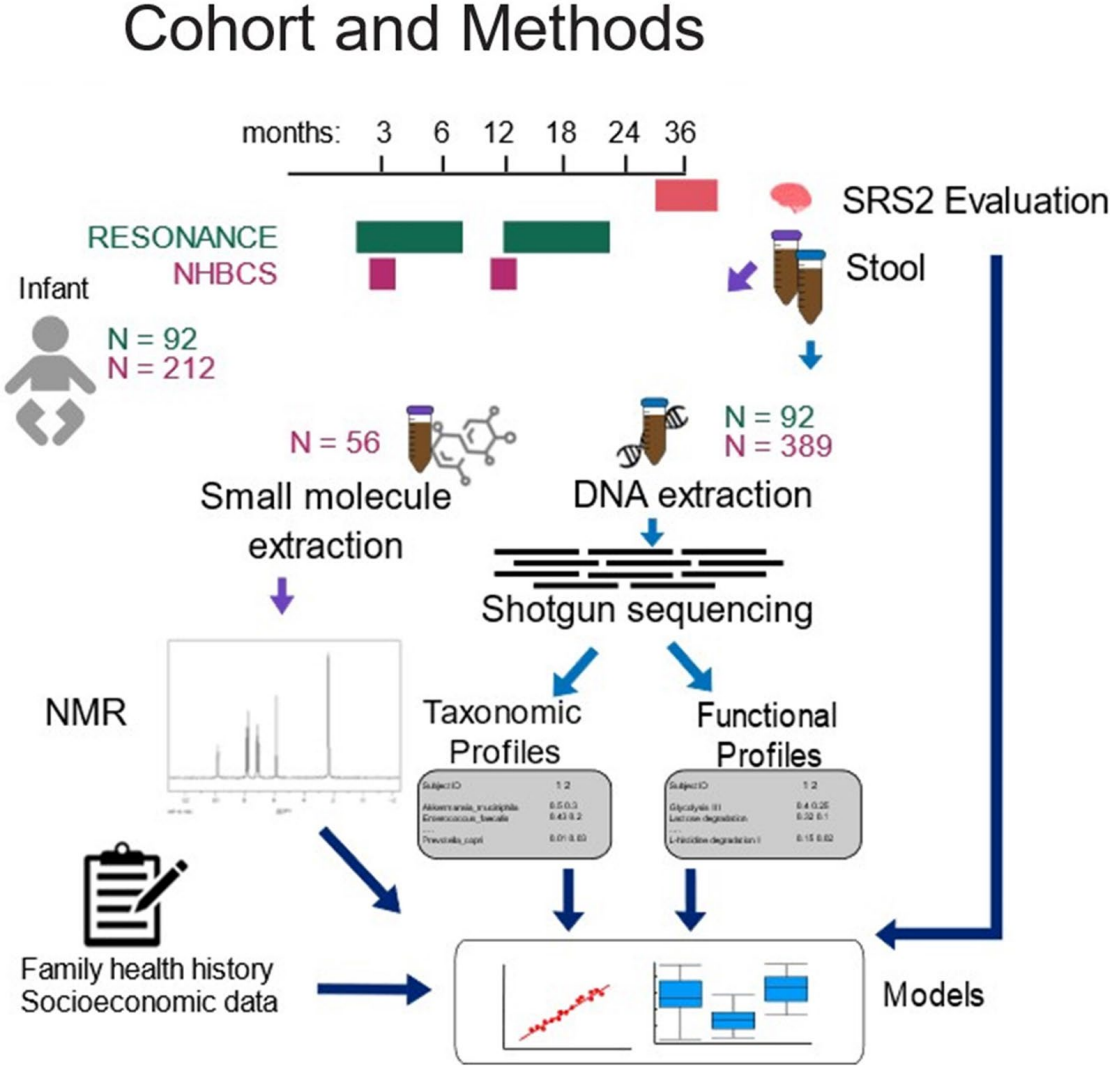
Diagram of Study Methods. Fecal samples were collected from RESONANCE participants between six weeks and two years postpartum and from New Hampshire Birth Cohort Study (NHBCS) participants at six weeks and/or one year. In both cohorts, DNA was extracted from stools and underwent shotgun metagenomic sequencing. In both cohorts, assessment of autism-spectrum disorder behaviors with the Social Responsiveness Scale, 2nd edition, was completed after age 3 years. Additionally, a subset of NHBCS fecal samples underwent nuclear magnetic resonance spectroscopy to quantify small molecules. These data were integrated with sociodemographic data into statistical models

An overview of the methods is presented in Fig. 1. The RESONANCE cohort and NHBCS are part of ECHO, a consortium that harmonizes data across cohorts to probe scientific questions regarding children’s environmental health that are unanswerable by a single cohort [27, 28]. Background on each of the cohorts, including eligibility criteria, is provided in the Supplemental Methods.

### Shotgun metagenomic sequencing

Fecal samples were collected in tubes containing an RNA stabilizer from a subset of child participants for microbiome sequencing and analysis. In the NHBCS, fecal samples (n = 389) were collected from 212 children at approximately 6 weeks and 12 months of age, stored in a polyethylene bag, and frozen in home freezers or directly transported on ice to maternal postnatal follow-up visits. Within 24 h of receipt, the samples were aliquoted and frozen at 80 °C. We restricted this analysis to samples collected before four years of age (n = 92). DNA was extracted from the stool using the Zymo DNA extraction kit (Zymo Research, Irvine, California) and prepared for metagenomic sequencing using Illumina NextSeq at Marine Biological Laboratory in Woods Hole, Massachusetts. In the RESONANCE cohort, stool samples were collected by parents in OMR-200 tubes (OMNIgene GUT, DNA Genotek, Ottawa, Ontario, Canada), immediately stored on ice, and brought within 24 h to the lab in Rhode Island where they were immediately frozen at − 80 °C. Stool samples were not collected if the subject had taken antibiotics within the last 2 weeks. DNA extraction was performed at Wellesley College (Wellesley, Massachusetts). Nucleic acids were extracted from stool samples using the Rneasy PowerMicrobiome kit automated on the QIAcube (Qiagen, Germantown, Maryland), excluding the DNA degradation steps. Shotgun metagenomic sequencing was performed on extracted DNA at the Integrated Microbiome Resource (IMR, Dalhousie University, Nova Scotia, Canada).

For the NHBCS, sequencing libraries were prepared using Nugen’s Ovation Ultralow V2 protocol. The extracted DNA samples were sheared to a mean insert size of 400 bp using a Covaris S220 focused ultrasonicator, resulting in an average of 30 million reads per sample. For the RESONANCE cohort, a pooled library (max 96 samples per run) was prepared using the Illumina Nextera Flex Kit for MiSeq and NextSeq from 1 ng of each sample. Samples were then pooled onto a plate and sequenced on the Illumina NextSeq 550 platform using 150 + 150 bp paired-end “high-output” chemistry, generating ~ 400 million raw reads and ~ 120 Gb of sequence per plate. For both cohorts, the BioBakery v3 pipeline was used to process the raw sequences [29]. KneadData (v0.7.7) was used to trim adapters from raw sequence reads and remove reads matching a human genome reference. Metagenomic Phylogenetic Analysis (MetaPhlAn v3.0.7) was used to generate taxonomic profiles by aligning reads to a reference database of marker genes (mpa_v30_CHOCOPhlAn_201901) [29]. Finally, the Human Microbiome Project Unified Metabolic Analysis Network (HUMAnN v3.0.0a4 with databases at v201901b) was used to functionally profile metagenomes [29] .

### Fecal metabolomics

In the NHBCS, stool samples were collected for metabolomics analysis in trace element free tubes and sent to the Eastern Regional Comprehensive Metabolomics Research Core at the Research Triangle Institute for nuclear magnetic resonance (NMR) analysis. NMR spectra of fecal samples were acquired on a Bruker 700 MHz NMR spectrometer (Billerica, Massachusetts). The relative concentrations of library-matched metabolites associated with host and gut microbe co-metabolism [30–32] were obtained using the Chenomx NMR Suite 8.1 Professional (Chenomx Inc., Edmonton, Alberta, Canada) [33, 34]. Relative concentrations were log_2_-transformed for analysis.

### Outcome assessment

Caregivers assessed the participant’s social skills using the Social Responsiveness Scale, 2nd edition (SRS-2), which captures ASD-related social behaviors in multiple domains, in both cohorts [35]. Standardized total T-scores have a mean (standard deviation, SD) of 50 (10), with higher scores indicative of social behaviors associated with ASD. In the NHBCS, caregivers completed the preschool version of the SRS-2 when participants were ~ 3 years of age and the school-age version when the participants were 5 years of age. If a preschool assessment was not available, the participant was included with their school-age score. In the RESONANCE cohort, caregivers completed the SRS-2 preschool version for participants 2.5–4 years of age and the school-age version for participants 4–19 years of age.

### Covariates

Covariates were selected a priori based on their potential to confound the association between the microbiome and SRS-2 scores or as predictors of the outcome. At recruitment, study staff implemented questionnaires to collect data on maternal smoking during pregnancy (any vs. none), parity (parous vs. nulliparous), parental age (continuous), and sociodemographic characteristics, including maternal education (any higher education vs. none) and marital status. Characteristics of the birth (delivery mode: C-section vs. vaginal, peripartum antibiotic exposure: any vs. none, gestational age at delivery: continuous, and child’s sex) were abstracted from medical records (NHBCS) or questionnaires (RESONANCE). Repeated questionnaires throughout childhood captured duration of breastfeeding (continuous) and breastfeeding status at the time of stool sample collection (exclusive vs. any formula). Exact age at SRS-2 implementation was calculated as the difference between the date of birth and the date of survey completion (continuous). To reduce selection bias and improve power, we imputed missing covariates using the *mice* R package [36–38]. Details are available in the Supplemental Methods.

### Statistical analysis

Because many NHBCS participants provided two stool samples, we analyzed early (< 6 month) and late (6 month–2 year) samples separately. Additionally, we examined NHBCS and RESONANCE samples separately. We tested the overall association between bacterial species or functions and SRS-2 scores with *adonis2*, a PERMANOVA method that allows for quantification of variable influence (R^2^) and significance (marginal *p*-value) in community structure (Bray–Curtis distances) [39–41]. A *p*-value less than 0.05 was considered significant. Bacterial diversity was quantified with Shannon and Inverse Simpson indices. We linearly regressed diversity against SRS-2 scores, adjusting for the previously mentioned covariates, considering a p-value less than 0.05 significant. We tested whether models built on data from the NHBCS could predict SRS-2 scores in the RESONANCE cohort using the caret package in R [42]. Briefly, we used the predict function in the stats R package to predict SRS-2 scores for RESONANCE observations based on their microbiome and covariates and the model constructed using data from the NHBCS. Functions from the caret package were then used to calculate correlations and errors between actual and predicted scores. To screen for bacterial species associated with SRS-2 scores, we used microbiome multivariable association with linear models (MaAsLin2), which models bacterial relative abundance as the outcome [43]. Associations with a false discovery rate (FDR) of q < 0.1 were modeled in linear regression with bacterial relative abundance predicting SRS-2 scores, adjusting for covariates. Given the sex-ratio differential of ASD presentation, we explored whether relationships between the gut microbiome and behavior were different in biological males and females with interaction models [44].

Potentially neuroactive microbial gene sets were acquired from Supplementary Dataset 1 from Valles-Colomer et al. [45] Kyoto Encyclopedia of Genes and Genomes (KEGG) Orthologs (KOs) from this dataset were mapped to UniRef90s using the mapping files included with HUMAnN3. Pearson correlations for each measure (SRS-2 scores or residuals from a complete linear model regressing SRS-2 scores against all covariates) and each UniRef90 gene family found in ≥ 4 subjects were calculated. We performed Mann–Whitney U tests (implemented in the HypothesisTests.jl package) on the correlations of each gene family in a neuroactive gene set against the correlations of all gene families not found in the gene set [46]. FDR q < 0.1 was the threshold for significance [47].

To test if the capacity of the gut microbiome to have neuroactive genes associated with SRS-2 scores related to the metabolic profiles of infants, we regressed SRS-2 scores against relative concentrations of fecal metabolites identified as significant from the Mann–Whitney U test that were also annotated in our dataset. Contribution of specific taxa to the relative abundance of neuroactive gene pathways was determined using stratified MetaPhlAn tables. All models were run with R (4.1.1), RStudio (v1.4.1717), and Julia (v1.6.1). Code and package versions are available at https://github.com/HEL548/ECHOSRS2.

## Results

### Population characteristics

ECHO participants contributing to the analysis from the New Hampshire Birth Cohort Study (NHBCS) and Rhode Island RESONANCE cohorts had similar distributions of most characteristics (Table 1). As expected, RESONANCE participants were older at SRS-2 completion (8 ± 2.7 years compared with 3.2 ± 0.4 in NHBCS). As previously described [12], social behaviors of NHBCS participants were rated as having behavior less indicative of ASD (43.6 ± 4.8) than the normative population (50 ± 10), whereas RESONANCE participants were rated similar to the normative population (49.9 ± 8.5). RESONANCE stool samples, which were collected from older participants (6 months–2 years) than the NHBCS stool samples (6 weeks–2 years), had higher Shannon diversity (2.9 ± 0.5) than the NHBCS samples (1.9 ± 0.7).

**Table 1:** Population characteristics of New Hampshire birth cohort study (NHBCS) and RESONANCE participants included in any analysis [Mean ± Standard Deviation (% Missing If Any) or n (%)]

	NHBCS participants (n = 212)	Resonance participants (n = 92)
Maternal age (years)	32.4 ± 4.2	31.3 ± 5.1
Paternal age (years)	33.9 ± 5.8 (2.4)	34 ± 6.3 (10.9)
Maternal smoking during pregnancy		
None	200 (94.3)	85 (92.4)
Any	9 (4.2)	5 (5.4)
Missing	3 (1.4)	2 (2.2)
Maternal education		
Any graduate	93 (43.9)	67 (72.8)
Less than graduate	113 (53.3)	25 (27.2)
Missing	6 (2.8)	0 (0)
Marital status		
Married	188 (88.7)	75 (81.5)
Unmarried	18 (8.5)	17 (18.5)
Missing	6 (2.8)	0 (0)
Parity		
Parous	104 (49.1)	69 (75)
Nulliparous	105 (49.5)	23 (25)
Missing	3 (1.4)	0 (0)
Delivery mode		
Vaginal	149 (70.3)	63 (68.5)
C-section	58 (27.4)	28 (30.4)
Missing	5 (2.4)	1 (1.1)
Breastfeeding at stool collection		
No	78 (36.8)	3 (3.3)
Yes	112 (52.8)	71 (77.2)
Missing	22 (10.4)	18 (19.6)
Peripartum antibiotics		
Any	112 (52.8)	0 (0)
None	95 (44.8)	92 (100)
Missing	5 (2.4)	0 (0)
Gestational age (weeks)	39.2 ± 1.7	38.5. ± 1.8
Child’s sex		
Male	119 (56.1)	52 (56.5)
Female	93 (43.9)	40 (43.5)
Child age at follow-up (years)	3.2 ± 0.4	8 ± 2.7
Social responsiveness scale T-score	43.6 ± 4.8	49.9 ± 8.5
Shannon Index	1.9 ± 0.7	2.9 ± 0.5

### Gut microbiome communities and SRS-2 scores

**Fig. 2:**
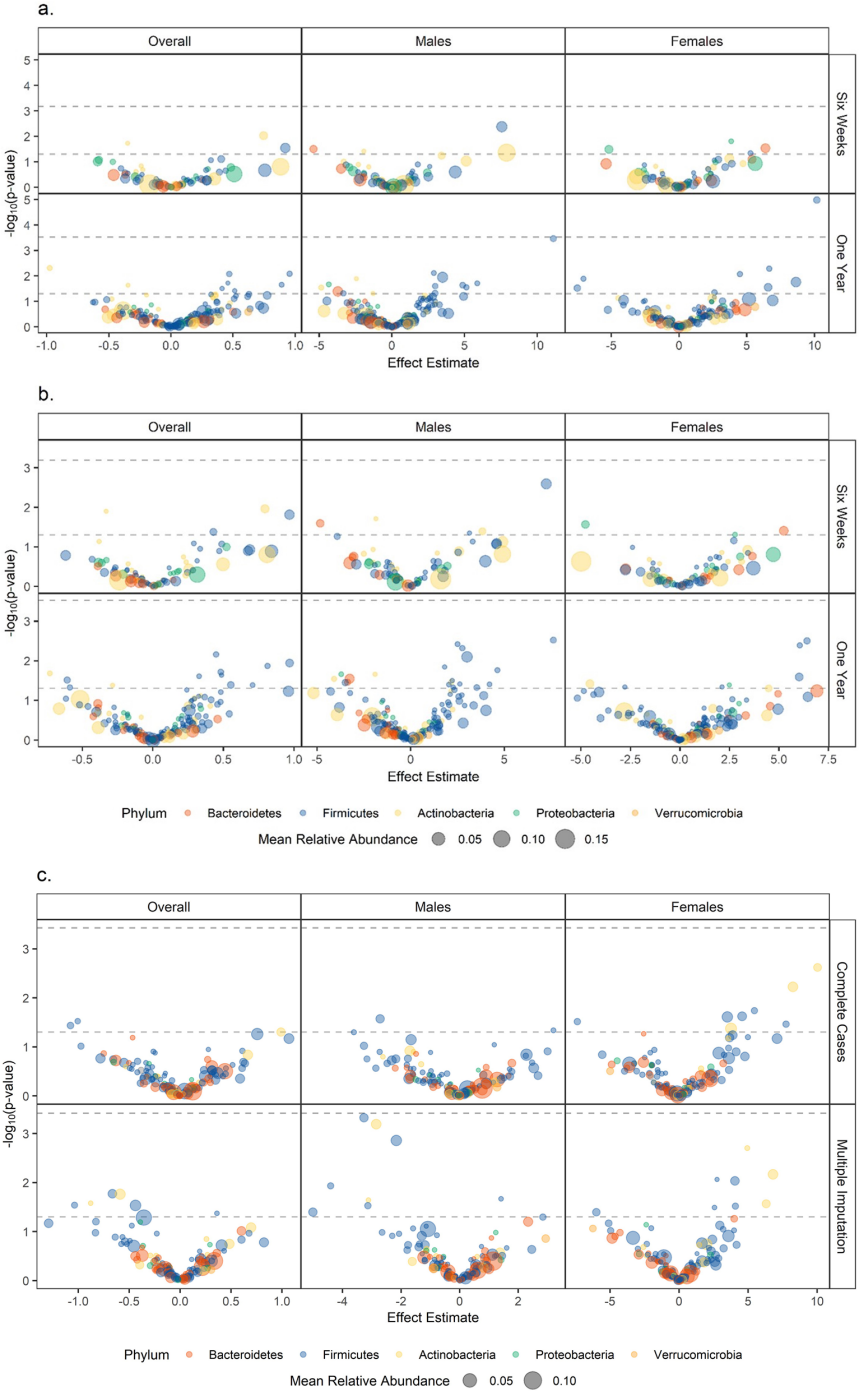
Associations Between Bacterial Species Relative Abundance and Social Responsiveness Scale, 2nd Edition (SRS-2), Scores. **A** Complete cases in the New Hampshire Birth Cohort Study (NHBCS). **B** Subjects with missing covariate data imputed in the NHBCS. **C** Among RESONANCE participants. Models adjusted for gestational age, child’s sex, peripartum antibiotic exposure, delivery mode, exclusive breastfeeding at the time of stool sample collection, maternal smoking during pregnancy, maternal and paternal age, maternal marital status, parity, maternal education, and the child’s age at SRS-2 completion. Each point reflects the association between a bacterial species and SRS-2 scores. Points are colored by phylum. Dashed gray lines indicate *p* = .05 and the Bonferroni corrected p-value for each analysis

Bray–Curtis differences in 6-week-old infants’ microbiota in the NHBCS contributed significantly to variability in SRS-2 scores (Additional file 1: Table S1, R^2^ = 0.01, *p* = 0.016). This association had a similar magnitude among males and females and was of borderline statistical significance in males (R^2^ = 0.008 *p* = 0.08), but not females (R^2^ = 0.006, *p* = 0.337). In contrast, microbial species in one-year-old infants did not significantly explain variability in SRS-2 scores (R^2^ = 0.006, *p* = 0.221). Microbial community structure in the RESONANCE cohort was marginally related to SRS-2 scores, explaining approximately 1.7% of the variability (*p* = 0.068). No association was observed between alpha diversity and SRS-2 scores, although increased diversity trended toward being associated with social behavior more indicative of ASD among girls (Additional file 1: Table S2). The models built on NHBCS data did not predict SRS-2 scores in the RESONANCE cohort (Additional file 1: Table S3). Most associations between bacterial species and SRS-2 scores did not reach statistical significance after adjusting for multiple comparisons (Fig. 2, Additional file 1: Table S4). Among females, higher relative abundance of *Enterococcus faecalis* at one year was associated with ASD-related social behaviors in the complete case analysis [β = 0.41 difference in SRS-2 score per doubling species relative abundance, 95% CI (0.17–0.65), *p* < 0.001, q = 0.002] whereas we observed no association among males [β = − 0.07, 95% CI (− 0.31–0.18), *p* = 0.6, q = 0.71, p_interaction_ = 0.006]. While this association was nominally significant in the multiple imputation analysis, the effect size was smaller among females and did not meet the threshold after adjusting for multiple comparisons [β = 0.23, 95% CI (0.01–0.14), q = 0.34]. Higher relative abundance of *Anaerostipes caccae* was associated with worse social behavior among males [β = 0.31, 95% CI (0.16–0.47), *p* < 0.001, q = 0.06] and nominally associated with better social behavior among females [β = − 0.2, 95% CI (− 0.37 to − 0.03), *p* = 0.02, q = 0.73, p_interaction_ < 0.001] in the complete case analysis. The association among males was nominally significant in the multiple imputation analysis, but the effect size was smaller [β = 0.27, 95% CI (0.11–0.42), *p* < 0.001] and did not meet the FDR threshold (q = 0.27). These associations were not replicable in the RESONANCE cohort due to low prevalence of these species (Fig. 2c).

### Functional potential of gut microorganisms

**Fig. 3:**
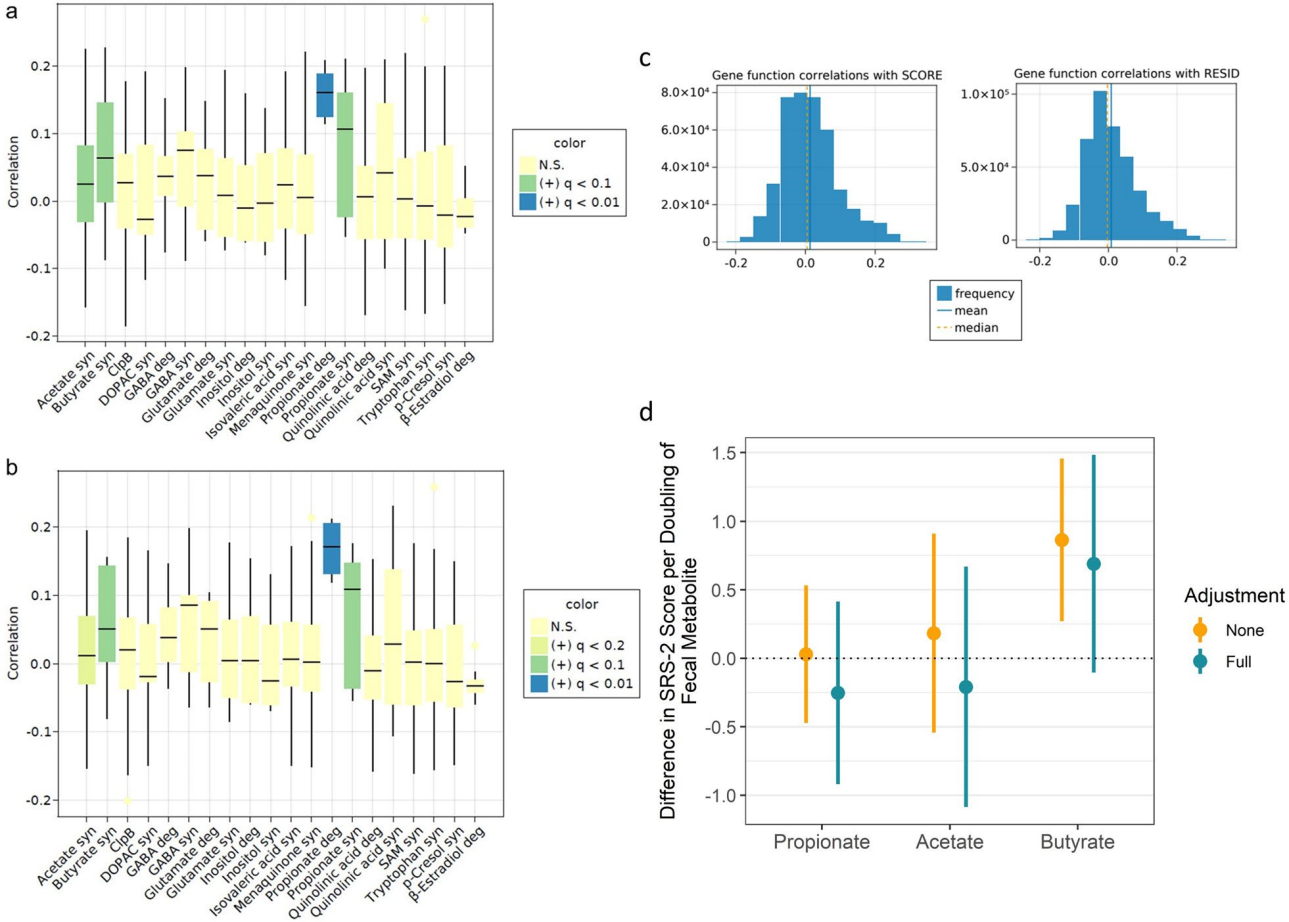
Associations of Microbial Functions and Social Responsiveness Scale, 2nd Edition (SRS-2) Scores. Feature Set Enrichment Analysis of **A** Raw SRS-2 scores and **B** regression model residuals with microbial gene sets. **C** Distribution of correlations between microbial gene families and SRS-2 scores (SCORE), and regression model residuals (RESID). **D** Association (point estimate and 95% confidence interval) between concentrations of fecal short-chain fatty acids and SRS-2 Scores in the Unadjusted and Fully Adjusted Models. Fully adjusted models include gestational age, child’s sex, peripartum antibiotic exposure, delivery mode, exclusive breastfeeding at the time of stool sample collection, maternal smoking during pregnancy, maternal and paternal age, maternal marital status, parity, maternal education, and the child’s age at SRS-2 completion

Differences in the functional capacity of gut bacteria in fecal samples collected at six weeks from NHBCS infants explained 1% of the variability in SRS-2 scores (Additional file 1: Table S1, *p* = 0.048) and the contribution of 1-year-old participants was similar (0.9%, *p* = 0.067). Feature set enrichment analysis (FSEA) demonstrated a strong association between genes encoding for SCFA synthesis at 6 weeks of age and ASD-related SRS-2 scores after multiple hypothesis correction, for both raw SRS-2 scores and linear model residuals (Fig. 3, Additional file 1: Table S5; butyrate synthesis SRS-2 scores, med = 0.064, model residuals: med = 0.050, both q = 0.066; acetate synthesis and SRS-2 scores: med = 0.025, q = 0.066, and model residuals: med = 0.012, q = 0.18). *Escherichia coli* was the largest contributor of butyrate synthesis genes in the NHBCS, determined from the species-stratified table of KO abundance. Interestingly, both synthesis and degradation of the 3-carbon SCFA propionate were significantly associated with ASD-related scores (synthesis, med = 0.107, q = 0.066; degradation, med = 0.161, q = 0.0047). Strikingly, this association was not observed in microbiome samples from older children (Additional file 1: Table S5). Synthesis of the branched SCFA isovaleric acid at 1 year of age was associated with more optimal social behaviors as measured by the SRS-2, but we did not observe any associations with the metabolism of butyrate, propionate, or acetate captured at this age. Rather, we observed a small, but very significant, association between the synthesis of menaquinone, or vitamin K2 (med = − 0.048, q < 0.001), and more optimal social behaviors. We utilized data from the RESONANCE cohort to validate these findings and observed the same association of propionate synthesis in infants (birth to 4 months of age) with autism-related behaviors in mid-childhood (med = 0.096, *p* = 0.037) and between menaquinone synthesis in older participants (age > 9 months) and more optimal social behaviors (med = − 0.070, *p* = 0.033). As expected, correlations between all gene families and each measure were approximately normally distributed (Fig. 3c).

### Fecal metabolites associated with SRS-2 scores

In the NHBCS, the relative concentrations of microbial-related metabolites, including propionate, acetate, and butyrate, were quantified in 6-week-old stool samples from 56 participants. Menaquinone and isovaleric acid were not annotated. Concentrations of acetate and propionate were not significantly associated with SRS-2 scores in unadjusted or adjusted models, whereas higher butyrate concentrations related to higher SRS-2 scores in unadjusted models [β = 0.86 point increase per doubling of butyrate, 95% CI (0.27–1.46), *p* = 0.006; Fig. 3d]. This association was attenuated and no longer significant in adjusted models [β = 0.69 point increase per doubling of butyrate, 95% CI (− 0.1–1.48), *p* = 0.097], likely due to a strong association between formula use and increased butyrate, including in NHBCS [34].

## Discussion

In this longitudinal analysis of the infant and toddler microbiome and social behaviors associated with ASD measured at preschool age, we found suggestive evidence that bacterial species, neuroactive bacterial functions, and metabolite patterns were prospectively associated with ASD-related traits. Our study adds to the existing body of research by examining the prospective development of the intestinal microbiome in early life in relation to subsequent autism-associated behaviors. The findings of this study highlight opportunities for identifying biomarkers for early detection and developing therapies for intervention and prevention of ASD.

We identified a significant association between the genes involved in the synthesis of specific SCFAs, particularly butyrate and acetate, in early life and ASD-related social behavior at approximately age 3. This signal was not evident at older ages, highlighting the importance of early-life microbiome factors in influencing later behavioral outcomes. Butyrate, in particular, has been linked to immune regulation and reduced inflammation, which are relevant to ASD [48]. It is important to note that we cannot directly infer gut concentrations of molecules based on the differences in gene abundances. Genes abundances reflect selective pressures encountered in the gut ecosystem and do not necessarily mean those genes are expressed. Further, low concentrations of SCFA in the gut may select for microbes that produce them, leading to a higher presence of SCFA synthesis genes.

To address this limitation, we augmented our analysis of the functional capacity of the gut microbiome with fecal metabolomic data in a subset of participants. These data supported our finding of an adverse association between butyrate and SRS-2 scores, but not acetate or propionate. Higher concentrations of butyrate, an essential energy source for colonic epithelial cells and gut barrier functions that modulate systemic inflammation [49], have been observed in children with clinical ASD < 5 years of age [50]. In one study, a paucity of *Bifidobacterium longum* in 2- to 4-year-olds with ASD, and an increase in *Faecalibacterium prausnitzii*, a known butyrate producer, were highlighted [50], suggesting that the substrates produced during the fermentation of nondigestible oligosaccharides by *Bifiidobacteria* and Ruminococcaceae are then utilized by butyrate producers such as *Faecalibacterium*, *Eubacterium*, *Roseburia*, and *Anaerostipes* [51]. Although *Anaerostipes hadrus* was a major contributor of butyrate synthesizing genes, *E. coli* provided the most butyrate synthesis genes in the NHBCS. This is likely due to the excellent characterization of the *E. coli* genome and its high prevalence in NHBCS infants. It is plausible that butyrate influences social behaviors through modulation of the excitatory/inhibitory balance, but the exact mechanism remains uncharacterized. Because both propionate synthesis and degradation pathways were adversely associated with SRS-2 scores, it is possible that the turnover of propionate rather than its concentration is significant to neurobehavior, or that SCFA concentrations must be in a “Goldilocks zone” requiring regulation of both synthesis and degradation [52]. Additionally, the concentration of microbial metabolites in feces, while proximal to their source, may not reflect circulating bioactive molecules in the host. Further research correlating the serum metabolome data with fecal metagenomes and metabolomes in very young children may provide additional insights into this association.

Increased capacity for menaquinone synthesis in older infants was associated with behavioral scores less indicative of ASD in both cohorts. Menaquinone may have anti-inflammatory and antioxidant effects in the brain [53], although it is unclear what proportion of host circulating menaquinone is bacterially-derived [54]. A successful probiotic intervention to relieve anxiety was shown to increase bacterial capacity for menaquinone production [55], among other metabolic changes, suggesting that bacterially-derived menaquinone may relate to better neurobehavioral health [56]. Additional metabolomic data could clarify our understanding of the importance of bacterial production of menaquinone in the human host.

## Limitations

While this study advances our understanding of the microbiome-autism relationship, it is not without limitations. Most NHBCS participants' social behavior was rated with the preschool-age form, whereas most RESONANCE participants were rated with the school-age form, potentially weakening the validation analysis. However, the use of T-scores reduces variation resulting from differences in the form. Furthermore, SRS-2 scores have high consistency over time, minimizing the effect of age at which the surveys were administered in the two cohorts [57]. Another limitation is the variation in stool sample collection methods between the two studies, including the timing of sample collection, which may have led to differential detection of certain species; much of the concern about collection and processing methods may be alleviated with a rigorous comparison study of methods identifying data alignment with variable methods [58].

Our study, while leveraging the resources and population of the ECHO consortium, had a limited sample size, including few participants who met the criteria for high risk of ASD. However, it reflects a population-based evaluation of neurodevelopment, which, when combined with case–control and mechanistic studies, adds valuable insight into features that alter subclinical symptoms of ASD. Our sex-specific analysis was likely underpowered, but given the known sex-specific differences in ASD prevalence, these findings should be replicated in another cohort. We incorporated exclusive human milk consumption and duration of human milk consumption in our models, but did not account for dietary patterns in old infants/toddlers. Further research involving clinical populations and animal models is needed to validate our findings and strengthen their applicability and translatability to clinical care.

## Conclusion

In conclusion, this study employed metagenomic sequencing data and advanced analysis methods to investigate the relationship between the infant microbiome and ASD-related behaviors. The use of FSEA allowed us to examine the metabolism of neuroactive molecules for associations with SRS-2 scores with more power than an untargeted pathway analysis. The validation set approach is novel in microbiome epidemiology and is crucial as the field advances. Leveraging the substantial resources of the ECHO consortium enabled this multicenter evaluation of two large US birth cohorts, providing a robust representation of the general population; this enabled an evaluation of the prospective risk of typical versus atypical neurodevelopment from the perspective of the gut-brain axis. This study demonstrated associations between SCFA synthesis, menaquinone production, and social behaviors in early life, suggesting potential avenues for early intervention and prevention. Our research contributes to the burgeoning research by beginning to clarifying the features of the microbiome that relate to subsequent behaviors prior to neuropsychiatric diagnoses. In this future, this will allow for research into interventions targeting the microbiome and changing neurodevelopmental trajectories.

### Supplementary Information

**Additional file 1**: Supplementary material contains the information about cohort characteristics and exclusion criteria. The following supplementary tables are included: Contribution of Bacterial Species and Functions to SRS-2 Scores determined with Bray-Curtis distances; Difference in SRS-2 Score per Standard Deviation Increase in Bacterial Species Diversity in the New Hampshire Birth Cohort Study; Validation of Diversity Models in RESONANCE Cohort with Multiple Imputation of Missing Covariates; Family Set Enrichment Analysis Results Among New Hampshire Birth Cohort Infants.

## Data Availability

The sequencing data used in this study are available through the National Center for Biotechnology Information (NCBI) Sequence Read Archive: https://ncbi.nlm.nih.gov/sra under accession numbers PRJNA296814 and PRJNA695570. Epidemiologic data are not publicly available due to their sensitive and identifiable nature, but are available from the corresponding author on reasonable request.

## Abbreviations

ASD: Autism spectrum disorder
ECHO: Environmental influences on children health outcomes
KEGG: Kyoto encyclopedia of genes and genomes
NHBCS: New Hampshire Birth Cohort Study
NMR: Nuclear magnetic resonance
SCFA: Short-chain fatty acid
SRS-2: Social Responsiveness Scale, 2nd edition
