# Supplementary material for: Prospective association of the infant gut microbiome with social behaviors in the ECHO consortium
Source: Mol Autism. 2024 May 17;15:21. doi: 10.1186/s13229-024-00597-2 (PMC11101342; doi:10.1186/s13229-024-00597-2)
Supplement: Supplementary file 1 — Additional file 1: Supplementary material contains the information about cohort characteristics and exclusion criteria. The following supplementary tables are included: Contribution of Bacterial Species and Functions to SRS-2 Scores determined with Bray-Curtis distances; Difference in SRS-2 Score per Standard Deviation Increase in Bacterial Species Diversity in the New Hampshire Birth Cohort Study; Validation of Diversity Models in RESONANCE Cohort with Multiple Imputation of Missing Covariates; Family Set Enrichment Analysis Results Among New Hampshire Birth Cohort Infants. [file 13229_2024_597_MOESM1_ESM.docx]

**Supplemental Methods**

**Cohort backgrounds**

NHBCS, initiated in 2009 and based at Dartmouth College in Hanover, New Hampshire (NH), is an ongoing prospective cohort study of over 2,500 pregnant women who received prenatal care from study clinics in NH and their offspring.^1^ The study was originally designed to examine the effects of drinking water and other contaminants on maternal-child health outcomes and therefore focused on enrolling pregnant women whose residence was served by a private, unregulated water system (eg, well water). Extensive environmental exposure and biospecimen data are available, as are assessments of neurodevelopmental cardiometabolic, and respiratory health outcomes. Demographic and clinical data are collected via questionnaires (eg, breastfeeding patterns) and medical records (e.g., peripartum antibiotic exposure). The RESONANCE cohort, initiated in 2010 and based at Brown University in Providence, Rhode Island, is an ongoing longitudinal observational study of healthy children that spans the fetal and infant to adolescent life stages, combining neuroimaging (magnetic resonance imaging, MRI), neurocognitive assessments, bio-specimen analyses, subject genetics, environmental exposures, and rich demographic, socioeconomic, family and medical history information.

**Cohort eligibility criteria**

For the New Hampshire Birth Cohort Study (NHBCS), eligible pregnancies included women (1) ages 25- 45 years, (2) who obtain routine prenatal care at participating study clinics in New Hampshire, (3) who have resided in the same place since their last menstrual period and have no plans to move prior to delivery, (4) who have a singleton pregnancy, and (5) who are English-speaking, literate, and mentally competent. For the RESONANCE cohort, the exclusion criteria are as follows: in utero exposure to alcohol, cigarettes or illicit substances; preterm (<37 weeks gestation) birth; small for gestational age or less than 1500 g; fetal ultrasound abnormalities; preeclampsia, high blood pressure, or gestational diabetes; 5-minute Apgar scores <8; neonatal intensive care unit (NICU) admission; neurological disorder (eg, head injury resulting in loss of consciousness, epilepsy); and psychiatric or learning disorder (including maternal depression) in the infant, parents, or siblings requiring medication in the year prior to pregnancy. Demographic and other non-biospecimen data such as race and ethnicity, parental education and occupation, feeding behavior (breast- and formula-feeding), and child weight and height, were collected through questionnaires or direct examination as appropriate. All data were collected at every assessment visit. All procedures for this study were approved by the local institutional review board at Rhode Island Hospital, and all experiments adhered to the regulation of the review board. Written informed consent was obtained from all parents or legal guardians of enrolled participants. Children in the RESONANCE cohort were born full-term (>37 weeks gestation) with height and weight normal for gestational age and from uncomplicated singleton pregnancies. Children with known major risk factors for developmental abnormalities at enrollment were excluded. In addition to screening at the time of enrollment, on-going screening for worrisome behaviors using validated tools was performed to identify at-risk children and remove them from subsequent analysis.

**Multiple imputation**

We imputed 40 datasets with 20 iterations each using predictive mean matching for continuous variables (paternal age) and logistic regression with bootstrapping for binary variables (delivery mode, exclusive breastfeeding at stool sample collection, maternal smoking during pregnancy, maternal education, marital status, parity, and peripartum antibiotic exposure). For linear regression models we utilized the *pool* function to combine estimates from separate regressions. For more complex models we constructed a single dataset from the 40 imputations using the subject-specific median for continuous variables and mode for categorical variables.

**References**

1. Madan JC, Hoen AG, Lundgren SN, et al. Association of Cesarean Delivery and Formula Supplementation With the Intestinal Microbiome of 6-Week-Old Infants. *JAMA pediatrics*. Mar 2016;170(3):212-9. doi:10.1001/jamapediatrics.2015.3732

**eTable 1. Contribution of Bacterial Species and Functions to SRS-2 Scores determined with Bray-Curtis distances**

| **Species or Functions** | **Imputed Covariates** | **Cohort** | **Age** | **Population** | **R^2^** | **p-value** | **q** |
| --- | --- | --- | --- | --- | --- | --- | --- |
| Species | No | NHBCS | Six Weeks | All | 0.011 | 0.036 | 0.54 |
| Species | No | NHBCS | Six Weeks | Boys | 0.007 | 0.318 | 1 |
| Species | No | NHBCS | Six Weeks | Girls | 0.009 | 0.103 | 1 |
| Species | No | NHBCS | One Year | All | 0.005 | 0.579 | 1 |
| Species | No | NHBCS | One Year | Boys | 0.006 | 0.376 | 1 |
| Species | No | NHBCS | One Year | Girls | 0.01 | 0.033 | 0.528 |
| Species | No | RESONANCE | All | All | 0.021 | 0.109 | 1 |
| Species | No | RESONANCE | All | Boys | 0.014 | 0.428 | 1 |
| Species | No | RESONANCE | All | Girls | 0.035 | 0.004 | 0.072 |
| Species | Yes | NHBCS | Six Weeks | All | 0.01 | 0.016 | 0.272 |
| Species | Yes | NHBCS | Six Weeks | Boys | 0.008 | 0.08 | 1 |
| Species | Yes | NHBCS | Six Weeks | Girls | 0.006 | 0.337 | 1 |
| Species | Yes | NHBCS | One Year | All | 0.006 | 0.221 | 1 |
| Species | Yes | NHBCS | One Year | Boys | 0.006 | 0.196 | 1 |
| Species | Yes | NHBCS | One Year | Girls | 0.007 | 0.089 | 1 |
| Species | Yes | RESONANCE | All | All | 0.017 | 0.068 | 0.952 |
| Species | Yes | RESONANCE | All | Boys | 0.013 | 0.248 | 1 |
| Species | Yes | RESONANCE | All | Girls | 0.016 | 0.106 | 1 |
| Functions | No | NHBCS | Six Weeks | All | 0.008 | 0.213 | 1 |
| Functions | No | NHBCS | Six Weeks | Boys | 0.006 | 0.438 | 1 |
| Functions | No | NHBCS | Six Weeks | Girls | 0.006 | 0.465 | 1 |
| Functions | No | NHBCS | One Year | All | 0.008 | 0.142 | 1 |
| Functions | No | NHBCS | One Year | Boys | 0.009 | 0.161 | 1 |
| Functions | No | NHBCS | One Year | Girls | 0.007 | 0.347 | 1 |
| Functions | No | RESONANCE | All | All |  |  |  |
| Functions | No | RESONANCE | All | Boys |  |  |  |
| Functions | No | RESONANCE | All | Girls |  |  |  |
| Functions | Yes | NHBCS | Six Weeks | All | 0.01 | 0.048 | 0.552 |
| Functions | Yes | NHBCS | Six Weeks | Boys | 0.008 | 0.12 | 1 |
| Functions | Yes | NHBCS | Six Weeks | Girls | 0.006 | 0.336 | 1 |
| Functions | Yes | NHBCS | One Year | All | 0.009 | 0.067 | 0.672 |
| Functions | Yes | NHBCS | One Year | Boys | 0.01 | 0.046 | 0.552 |
| Functions | Yes | NHBCS | One Year | Girls | 0.006 | 0.294 | 1 |
| Functions | Yes | RESONANCE | All | All |  |  |  |
| Functions | Yes | RESONANCE | All | Boys |  |  |  |
| Functions | Yes | RESONANCE | All | Girls |  |  |  |

**eTable 2. Difference in SRS-2 Score per Standard Deviation Increase in Bacterial Species Diversity in the New Hampshire Birth Cohort Study**

| **Index** | **Imputed Covariates** | **Age** | **Population** | **Estimate (95% CI)** | **p** |
| --- | --- | --- | --- | --- | --- |
| Inverse Simpson | No | Six Weeks | All | 0.26 (-0.5, 1.02) | 0.5 |
| Inverse Simpson | No | Six Weeks | Boys | -0.22 (-1.21, 0.76) | 0.66 |
| Inverse Simpson | No | Six Weeks | Girls | 0.95 (-0.22, 2.12) | 0.12 |
| Inverse Simpson | No | One Year | All | 0.17 (-0.55, 0.89) | 0.65 |
| Inverse Simpson | No | One Year | Boys | -0.04 (-1.04, 0.96) | 0.94 |
| Inverse Simpson | No | One Year | Girls | 0.4 (-0.65, 1.45) | 0.46 |
| Shannon | No | Six Weeks | All | 0.34 (-0.43, 1.11) | 0.38 |
| Shannon | No | Six Weeks | Boys | -0.04 (-1.04, 0.97) | 0.94 |
| Shannon | No | Six Weeks | Girls | 0.85 (-0.3, 2.01) | 0.15 |
| Shannon | No | One Year | All | 0.47 (-0.25, 1.19) | 0.2 |
| Shannon | No | One Year | Boys | 0.22 (-0.79, 1.22) | 0.67 |
| Shannon | No | One Year | Girls | 0.73 (-0.29, 1.76) | 0.16 |
| Inverse Simpson | Yes | Six Weeks | All | 0.28 (-0.43, 0.99) | 0.44 |
| Inverse Simpson | Yes | Six Weeks | Boys | -0.03 (-0.95, 0.9) | 0.95 |
| Inverse Simpson | Yes | Six Weeks | Girls | 0.71 (-0.38, 1.81) | 0.2 |
| Inverse Simpson | Yes | One Year | All | 0.28 (-0.43, 0.99) | 0.44 |
| Inverse Simpson | Yes | One Year | Boys | -0.04 (-0.94, 0.86) | 0.93 |
| Inverse Simpson | Yes | One Year | Girls | 0.32 (-0.68, 1.31) | 0.54 |
| Shannon | Yes | Six Weeks | All | 0.36 (-0.36, 1.08) | 0.32 |
| Shannon | Yes | Six Weeks | Boys | 0.16 (-0.78, 1.09) | 0.75 |
| Shannon | Yes | Six Weeks | Girls | 0.65 (-0.44, 1.74) | 0.25 |
| Shannon | Yes | One Year | All | 0.23 (-0.43, 0.89) | 0.49 |
| Shannon | Yes | One Year | Boys | 0.02 (-0.91, 0.95) | 0.97 |
| Shannon | Yes | One Year | Girls | 0.46 (-0.5, 1.42) | 0.35 |

**eTable 3. Validation of Diversity Models in RESONANCE Cohort with Multiple Imputation of Missing Covariates**

| **Index** | **Model** | **R^2^** | **RMSE**^a^ | **MAE**^b^ |
| --- | --- | --- | --- | --- |
| Inverse Simpson | Six Week Overall | 0.04 | 3164.82 | 3001.49 |
| Inverse Simpson | Six Week Sex-specific | 0.04 | 2784.45 | 2640.72 |
| Inverse Simpson | One Year Overall | 0.04 | 5320.63 | 5046.19 |
| Inverse Simpson | One Year Sex-specific | 0.04 | 5279.56 | 5007.24 |
| Shannon | Six Week Overall | 0.04 | 3187.69 | 3023.18 |
| Shannon | Six Week Sex-specific | 0.04 | 2972.03 | 2818.54 |
| Shannon | One Year Overall | 0.04 | 5381.69 | 5104.1 |
| Shannon | One Year Sex-specific | 0.04 | 5300.46 | 5027.06 |

^a^ Root mean square error

^b^ Mean absolute error

**eTable 4. Bacterial Species Associated with Social Responsiveness Scale Scores in the New Hampshire Birth Cohort Study**

| Imputed Covariates | Population | Age at Stool Sample | Species | Effect Estimate | Standard Deviation | p-value | q-value | Sex-interaction p-value |
| --- | --- | --- | --- | --- | --- | --- | --- | --- |
| No | All | Six weeks | *Enterococcus faecalis* | -0.02 | 0.06 | 0.738 | 0.939 | - |
| No | Male | Six weeks | *Enterococcus faecalis* | 0.001 | 0.08 | 0.985 | 0.998 | 0.689 |
| No | Female | Six weeks | *Enterococcus faecalis* | -0.05 | 0.09 | 0.602 | 0.950 |  |
| No | All | Six weeks | *Anaerostipes caccae* | Did not have high enough prevalence for analysis. | | | | |
| No | Male | Six weeks | *Anaerostipes caccae* |  |  |  |  |  |
| No | Female | Six weeks | *Anaerostipes caccae* |  |  |  |  |  |
| Yes | All | Six weeks | *Enterococcus faecalis* | 0.02 | 0.06 | 0.743 | 0.949 | - |
| Yes | Male | Six weeks | *Enterococcus faecalis* | 0.03 | 0.08 | 0.732 | 0.874 | 0.885 |
| Yes | Female | Six weeks | *Enterococcus faecalis* | 0.01 | 0.08 | 0.909 | 0.956 |  |
| Yes | All | Six weeks | *Anaerostipes caccae* | Did not have high enough prevalence for analysis. | | | | |
| Yes | Male | Six weeks | *Anaerostipes caccae* |  |  |  |  |  |
| Yes | Female | Six weeks | *Anaerostipes caccae* |  |  |  |  |  |
| No | All | One Year | *Enterococcus faecalis* | 0.17 | 0.09 | 0.053 | 0.673 | - |
| No | Male | One Year | *Enterococcus faecalis* | -0.07 | 0.12 | 0.598 | 0.714 | 0.006 |
| No | Female | One Year | *Enterococcus faecalis* | 0.41 | 0.12 | 9.9E-04 | 0.002 |  |
| No | All | One Year | *Anaerostipes caccae* | 0.08 | 0.06 | 0.196 | 0.780 | - |
| No | Male | One Year | *Anaerostipes caccae* | 0.31 | 0.08 | 8.5E-05 | 0.057 | 1.6E-05 |
| No | Female | One Year | *Anaerostipes caccae* | -0.20 | 0.08 | 0.018 | 0.729 |  |
| Yes | All | One Year | *Enterococcus faecalis* | 0.08 | 0.08 | 0.362 | 0.910 | - |
| Yes | Male | One Year | *Enterococcus faecalis* | -0.09 | 0.12 | 0.439 | 0.765 | 0.043 |
| Yes | Female | One Year | *Enterococcus faecalis* | 0.23 | 0.11 | 0.0395 | 0.343 |  |
| Yes | All | One Year | *Anaerostipes caccae* | 0.07 | 0.06 | 0.216 | 0.806 | - |
| Yes | Male | One Year | *Anaerostipes caccae* | 0.27 | 0.08 | 4.5E-04 | 0.272 | 1.3E-04 |
| Yes | Female | One Year | *Anaerostipes caccae* | -0.17 | 0.08 | 0.042 | 0.918 |  |

**eTable 5.** **Family Set Enrichment Analysis Results Among New Hampshire Birth Cohort Infants**

| **Age** | **Gene Set** | **Variable**^a^ | **Median** | **U** | **f** | **pvalue** | **qvalue** | **allmed** |
| --- | --- | --- | --- | --- | --- | --- | --- | --- |
| Six Weeks | Propionate degradation | RESID | 0.171 | 2.36E+06 | 0.952 | 0.000 | 0.003 | -0.003 |
| Six Weeks | Propionate degradation | SCORE | 0.161 | 2.31E+06 | 0.933 | 0.000 | 0.004 | 0.004 |
| Six Weeks | Acetate synthesis | SCORE | 0.025 | 1.77E+07 | 0.586 | 0.010 | 0.086 | 0.004 |
| Six Weeks | Propionate synthesis | SCORE | 0.107 | 3.82E+06 | 0.712 | 0.008 | 0.086 | 0.004 |
| Six Weeks | Butyrate synthesis | SCORE | 0.064 | 4.08E+06 | 0.705 | 0.007 | 0.086 | 0.004 |
| Six Weeks | Propionate synthesis | RESID | 0.108 | 3.79E+06 | 0.705 | 0.010 | 0.086 | -0.003 |
| Six Weeks | Butyrate synthesis | RESID | 0.050 | 4.02E+06 | 0.694 | 0.011 | 0.086 | -0.003 |
| Six Weeks | Acetate synthesis | RESID | 0.011 | 1.72E+07 | 0.570 | 0.036 | 0.167 | -0.003 |
| Six Weeks | GABA synthesis | RESID | 0.085 | 3.02E+06 | 0.665 | 0.056 | 0.227 | -0.003 |
| Six Weeks | GABA synthesis | SCORE | 0.075 | 2.92E+06 | 0.643 | 0.099 | 0.331 | 0.004 |
| Six Weeks | 17-beta-Estradiol degradation | RESID | -0.032 | 1.28E+06 | 0.344 | 0.106 | 0.335 | -0.003 |
| Six Weeks | Quinolinic acid synthesis | SCORE | 0.041 | 3.76E+06 | 0.607 | 0.149 | 0.447 | 0.004 |
| Six Weeks | Isovaleric acid synthesis | RESID | 0.006 | 8.19E+06 | 0.566 | 0.173 | 0.495 | -0.003 |
| Six Weeks | Quinolinic acid synthesis | RESID | 0.028 | 3.67E+06 | 0.593 | 0.211 | 0.577 | -0.003 |
| Six Weeks | Tryptophan synthesis | SCORE | -0.006 | 1.78E+07 | 0.468 | 0.299 | 0.660 | 0.004 |
| Six Weeks | GABA degradation | RESID | 0.038 | 1.08E+06 | 0.653 | 0.287 | 0.660 | -0.003 |
| Six Weeks | Glutamate degradation | RESID | 0.050 | 2.22E+06 | 0.598 | 0.308 | 0.660 | -0.003 |
| Six Weeks | ClpB (ATP-dependent chaperone protein) | SCORE | 0.027 | 3.95E+06 | 0.562 | 0.371 | 0.680 | 0.004 |
| Six Weeks | 17-beta-Estradiol degradation | SCORE | -0.023 | 1.55E+06 | 0.416 | 0.384 | 0.680 | 0.004 |
| Six Weeks | Isovaleric acid synthesis | SCORE | 0.024 | 7.88E+06 | 0.545 | 0.354 | 0.680 | 0.004 |
| Six Weeks | Glutamate degradation | SCORE | 0.038 | 2.19E+06 | 0.587 | 0.361 | 0.680 | 0.004 |
| Six Weeks | Menaquinone synthesis | RESID | 0.002 | 1.74E+07 | 0.527 | 0.392 | 0.680 | -0.003 |
| Six Weeks | Glutamate synthesis | SCORE | 0.008 | 8.43E+06 | 0.536 | 0.430 | 0.716 | 0.004 |
| Six Weeks | Tryptophan synthesis | RESID | 0.000 | 1.82E+07 | 0.479 | 0.501 | 0.771 | -0.003 |
| Six Weeks | Glutamate synthesis | RESID | 0.004 | 8.35E+06 | 0.532 | 0.493 | 0.771 | -0.003 |
| Six Weeks | ClpB (ATP-dependent chaperone protein) | RESID | 0.020 | 3.83E+06 | 0.545 | 0.516 | 0.774 | -0.003 |
| Six Weeks | GABA degradation | SCORE | 0.036 | 969775 | 0.586 | 0.546 | 0.779 | 0.004 |
| Six Weeks | Quinolinic acid degradation | SCORE | 0.006 | 1.00E+07 | 0.476 | 0.558 | 0.779 | 0.004 |
| Six Weeks | Menaquinone synthesis | SCORE | 0.005 | 1.71E+07 | 0.518 | 0.571 | 0.779 | 0.004 |
| Six Weeks | p-Cresol synthesis | RESID | -0.025 | 1.88E+06 | 0.455 | 0.627 | 0.818 | -0.003 |
| Six Weeks | Quinolinic acid degradation | RESID | -0.010 | 1.01E+07 | 0.481 | 0.647 | 0.826 | -0.003 |
| Six Weeks | p-Cresol synthesis | SCORE | -0.020 | 1.93E+06 | 0.466 | 0.715 | 0.875 | 0.004 |
| Six Weeks | Inositol synthesis | RESID | -0.025 | 1.34E+06 | 0.464 | 0.744 | 0.875 | -0.003 |
| Six Weeks | Inositol degradation | SCORE | -0.009 | 1.57E+06 | 0.474 | 0.798 | 0.908 | 0.004 |
| Six Weeks | DOPAC synthesis | RESID | -0.019 | 2.16E+06 | 0.522 | 0.802 | 0.908 | -0.003 |
| Six Weeks | DOPAC synthesis | SCORE | -0.026 | 2.04E+06 | 0.494 | 0.952 | 0.952 | 0.004 |
| Six Weeks | SAM synthesis | SCORE | 0.003 | 5.73E+06 | 0.495 | 0.931 | 0.952 | 0.004 |
| Six Weeks | Inositol synthesis | SCORE | -0.002 | 1.40E+06 | 0.485 | 0.893 | 0.952 | 0.004 |
| Six Weeks | SAM synthesis | RESID | 0.002 | 5.74E+06 | 0.496 | 0.949 | 0.952 | -0.003 |
| Six Weeks | Inositol degradation | RESID | 0.004 | 1.70E+06 | 0.514 | 0.885 | 0.952 | -0.003 |
| One Year | Menaquinone synthesis | SCORE | -0.046 | 1.25E+07 | 0.366 | 3.35E-05 | 0.000 | -0.013 |
| One Year | Menaquinone synthesis | RESID | -0.048 | 1.25E+07 | 0.365 | 2.95E-05 | 0.000 | -0.013 |
| One Year | Isovaleric acid synthesis | SCORE | -0.045 | 5.68E+06 | 0.384 | 0.018 | 0.180 | -0.013 |
| One Year | Isovaleric acid synthesis | RESID | -0.055 | 5.66E+06 | 0.383 | 0.017 | 0.180 | -0.013 |
| One Year | Tryptophan synthesis | RESID | -0.025 | 1.74E+07 | 0.444 | 0.061 | 0.442 | -0.013 |
| One Year | ClpB (ATP-dependent chaperone protein) | RESID | -0.041 | 2.70E+06 | 0.376 | 0.077 | 0.442 | -0.013 |
| One Year | Propionate synthesis | RESID | -0.036 | 1.94E+06 | 0.353 | 0.067 | 0.442 | -0.013 |
| One Year | Inositol synthesis | SCORE | -0.053 | 953362 | 0.323 | 0.104 | 0.469 | -0.013 |
| One Year | Propionate synthesis | SCORE | -0.045 | 2.03E+06 | 0.370 | 0.105 | 0.469 | -0.013 |
| One Year | SAM synthesis | RESID | -0.028 | 4.91E+06 | 0.416 | 0.124 | 0.485 | -0.013 |
| One Year | Inositol synthesis | RESID | -0.031 | 992491 | 0.336 | 0.133 | 0.485 | -0.013 |
| One Year | SAM synthesis | SCORE | -0.038 | 4.99E+06 | 0.422 | 0.155 | 0.516 | -0.013 |
| One Year | Tryptophan synthesis | SCORE | -0.016 | 1.80E+07 | 0.460 | 0.181 | 0.533 | -0.013 |
| One Year | Acetate synthesis | RESID | -0.018 | 1.41E+07 | 0.456 | 0.200 | 0.533 | -0.013 |
| One Year | Propionate degradation | RESID | -0.035 | 873606 | 0.345 | 0.189 | 0.533 | -0.013 |
| One Year | Propionate degradation | SCORE | -0.027 | 926536 | 0.366 | 0.256 | 0.641 | -0.013 |
| One Year | GABA synthesis | SCORE | -0.018 | 1.89E+06 | 0.406 | 0.284 | 0.654 | -0.013 |
| One Year | DOPAC synthesis | SCORE | -0.049 | 1.76E+06 | 0.417 | 0.367 | 0.654 | -0.013 |
| One Year | ClpB (ATP-dependent chaperone protein) | SCORE | -0.033 | 3.11E+06 | 0.433 | 0.345 | 0.654 | -0.013 |
| One Year | Acetate synthesis | SCORE | -0.019 | 1.44E+07 | 0.468 | 0.343 | 0.654 | -0.013 |
| One Year | GABA synthesis | RESID | -0.002 | 1.93E+06 | 0.415 | 0.329 | 0.654 | -0.013 |
| One Year | DOPAC synthesis | RESID | -0.048 | 1.77E+06 | 0.419 | 0.376 | 0.654 | -0.013 |
| One Year | Butyrate synthesis | RESID | -0.015 | 2.71E+06 | 0.428 | 0.334 | 0.654 | -0.013 |
| One Year | Butyrate synthesis | SCORE | -0.014 | 2.78E+06 | 0.440 | 0.421 | 0.701 | -0.013 |
| One Year | Inositol degradation | SCORE | -0.017 | 1.46E+06 | 0.433 | 0.516 | 0.802 | -0.013 |
| One Year | Glutamate degradation | SCORE | 0.017 | 2.13E+06 | 0.561 | 0.521 | 0.802 | -0.013 |
| One Year | Glutamate synthesis | SCORE | -0.019 | 8.35E+06 | 0.508 | 0.860 | 0.910 | -0.013 |
| One Year | p-Cresol synthesis | SCORE | -0.016 | 2.01E+06 | 0.475 | 0.792 | 0.910 | -0.013 |
| One Year | GABA degradation | SCORE | -0.012 | 799249 | 0.473 | 0.856 | 0.910 | -0.013 |
| One Year | Quinolinic acid synthesis | SCORE | -0.022 | 3.34E+06 | 0.527 | 0.713 | 0.910 | -0.013 |
| One Year | Quinolinic acid degradation | SCORE | -0.018 | 1.12E+07 | 0.510 | 0.786 | 0.910 | -0.013 |
| One Year | Glutamate synthesis | RESID | -0.002 | 7.96E+06 | 0.484 | 0.735 | 0.910 | -0.013 |
| One Year | p-Cresol synthesis | RESID | 0.002 | 2.01E+06 | 0.475 | 0.789 | 0.910 | -0.013 |
| One Year | GABA degradation | RESID | 0.003 | 928143 | 0.550 | 0.727 | 0.910 | -0.013 |
| One Year | 17-beta-Estradiol degradation | RESID | -0.031 | 2.02E+06 | 0.480 | 0.827 | 0.910 | -0.013 |
| One Year | Quinolinic acid synthesis | RESID | 0.002 | 3.39E+06 | 0.535 | 0.631 | 0.910 | -0.013 |
| One Year | Inositol degradation | RESID | 0.006 | 1.56E+06 | 0.462 | 0.713 | 0.910 | -0.013 |
| One Year | Glutamate degradation | RESID | -0.001 | 1.96E+06 | 0.516 | 0.865 | 0.910 | -0.013 |
| One Year | 17-beta-Estradiol degradation | SCORE | -0.041 | 2.06E+06 | 0.487 | 0.893 | 0.916 | -0.013 |
| One Year | Quinolinic acid degradation | RESID | -0.010 | 1.10E+07 | 0.500 | 0.984 | 0.984 | -0.013 |

^a^ SCORE: SRS-T scores were used in the FSEA analysis. RESID: Covariates were regressed against SRS-2 scores and the residuals were used in the FSEA analysis.
